# Trends and determinants of stillbirth in developing countries: results from the Global Network’s Population-Based Birth Registry

**DOI:** 10.1186/s12978-018-0526-3

**Published:** 2018-06-22

**Authors:** Sarah Saleem, Shiyam Sunder Tikmani, Elizabeth M. McClure, Janet L. Moore, Syed Iqbal Azam, Sangappa M. Dhaded, Shivaprasad S. Goudar, Ana Garces, Lester Figueroa, Irene Marete, Constance Tenge, Fabian Esamai, Archana B. Patel, Sumera Aziz Ali, Farnaz Naqvi, Musaku Mwenchanya, Elwyn Chomba, Waldemar A. Carlo, Richard J. Derman, Patricia L. Hibberd, Sherri Bucher, Edward A. Liechty, Nancy Krebs, K. Michael Hambidge, Dennis D. Wallace, Marion Koso-Thomas, Menachem Miodovnik, Robert L. Goldenberg

**Affiliations:** 1 Department of Community Health Sciences, Aga Khan University, Karachi, Pakistan; 2 RTI International, Durham, NC USA; 3 KLE Academy of Higher Education and Research, J N Medical College Belgaum, Karnataka, India; 4 INCAP, Guatemala City, Guatemala; 5 Moi University School of Medicine, Eldoret, Kenya; 6 Lata Medical Research Foundation, Nagpur, India; 7 University of Zambia, Lusaka, Zambia; 8 University of Alabama at Birmingham, Birmingham, AL USA; 9 Thomas Jefferson University, Philadelphia, PA USA; 10 Boston University, Boston, MA USA; 11 Indiana University, Indianapolis, IN USA; 12 University of Colorado, Denver, CO USA; 13 NICHD, Bethesda, MD USA; 14 Columbia University Medical Center, New York, NY USA

**Keywords:** Stillbirth, Low-middle income countries, Rates of decline

## Abstract

**Background:**

Stillbirth rates remain high, especially in low and middle-income countries, where rates are 25 per 1000, ten-fold higher than in high-income countries. The United Nations’ Every Newborn Action Plan has set a goal of 12 stillbirths per 1000 births by 2030 for all countries.

**Methods:**

From a population-based pregnancy outcome registry, including data from 2010 to 2016 from two sites each in Africa (Zambia and Kenya) and India (Nagpur and Belagavi), as well as sites in Pakistan and Guatemala, we evaluated the stillbirth rates and rates of annual decline as well as risk factors for 427,111 births of which 12,181 were stillbirths.

**Results:**

The mean stillbirth rates for the sites were 21.3 per 1000 births for Africa, 25.3 per 1000 births for India, 56.9 per 1000 births for Pakistan and 19.9 per 1000 births for Guatemala. From 2010 to 2016, across all sites, the mean stillbirth rate declined from 31.7 per 1000 births to 26.4 per 1000 births for an average annual decline of 3.0%. Risk factors for stillbirth were similar across the sites and included maternal age < 20 years and age > 35 years. Compared to parity 1–2, zero parity and parity > 3 were both associated with increased stillbirth risk and compared to women with any prenatal care, women with no prenatal care had significantly increased risk of stillbirth in all sites.

**Conclusions:**

At the current rates of decline, stillbirth rates in these sites will not reach the Every Newborn Action Plan goal of 12 per 1000 births by 2030. More attention to the risk factors and treating the causes of stillbirths will be required to reach the Every Newborn Action Plan goal of stillbirth reduction.

**Trial registration:**

NCT01073475.

## Background

For international comparisons, the World Health Organization (WHO) defines a stillbirth as a baby born with no signs of life at or after 28 weeks’ gestation [1]. Globally in 2015, 2.6 million third trimester stillbirths occurred and most of these were in low- and middle-income countries (LMICs), three quarters in south Asia and sub-Saharan Africa [2]. The third trimester stillbirth rate in south Asia and sub-Saharan Africa is approximately 10 times that of developed countries (29 vs. 3 per 1000 births) [2]. The number of stillbirths that occur in the second trimester in LMICs is unknown but in high-income countries, half of all stillbirths occur between 20 and 28 weeks of gestation [2].

Several distal, intermediate and proximal factors contribute to the high stillbirth rates in LMIC, and these tend to be related to one another [3]. Potential distal factors include lack of education of women, low socioeconomic status, and the inability to make timely decisions about seeking care. Intermediate factors related to stillbirth may include advanced or young maternal age, lack of awareness about danger signs, delay in moving to a hospital, non-availability of community resources, and poor maternal nutritional status [4, 5]. Finally, maternal and fetal medical conditions and the poor response of the health care system to these conditions act as proximal risk factors for stillbirth. As an example of the inter-relatedness of these factors, illiteracy is often associated with poverty, which is correlated with food insecurity, malnutrition and anemia. Poverty and low educational status can also affect a family’s decisions about seeking care such as identifying danger signs, and accessing antenatal, delivery, or emergency care. In many resource-poor countries, even when women may reach a facility in time for a potentially life-saving intervention, inadequately prepared facilities may fail to prevent adverse maternal or fetal outcomes [6].

From a public health perspective, and especially for LMICs, much of the literature on maternal mortality has emphasized determining a clinical cause for deaths occurring in hospitals and community settings. In addition, the three-delay model has been used to address distal and proximate determinants of maternal mortality and to test treatment protocols for well-defined clinical causes of maternal death.

However, until recently, this interest was lacking for fetal deaths as well as for perinatal and early neonatal mortality. Even though recent global analyses suggest a 25.5% overall decline in stillbirth rates over the last 5 years, large variations in stillbirth rates exist between and within LMIC, and many LMICs have experienced little if any reduction in stillbirths [7, 8]. The majority of these fetal deaths occur in the intra-partum period and most of these occur at or near term and many are associated with maternal causes of morbidity and mortality. However, in two-thirds of these stillbirths there was no indication of a maternal complication prior to going into labor [2].

The available literature suggests that several risk factors for stillbirth are common across LMICs [3]. These risk factors may vary by country depending upon the availability of resources for provision of care, as well as access to care by remotely located populations. The lack of a functional vital registration system also appears to be associated with high stillbirth rates [2, 7]. National data for stillbirth rates are rarely available from countries where most stillbirths occur. In LMICs, demographic and health surveys generally have not included stillbirths as routine pregnancy outcomes, and when this information is available, it is subjective and based on the responses of women without validation of gestational age [3, 9]. In many countries, information on stillbirth is derived primarily from hospital-based settings or based on data collected from a small number of locations.

We have previously explored stillbirth rates in sites participating in the Global Network for Women’s and Children’s Health Research [10–12]. The objective of this paper is to update trends in stillbirth rates over the years at these sites and to estimate the average annual percent change in these rates. In addition, we wanted to determine and compare distal and intermediate factors associated with stillbirths in sites in Africa, Pakistan, India and Guatemala.

## Methods

The Global Network’s Maternal Newborn Health Registry (MNHR) is a prospective, population-based observational study that includes all pregnant women and their outcomes in defined geographic communities (clusters). These clusters were established with approximately 300–500 annual births in sites in western Kenya, Zambia (Kafue and Chongwe), Pakistan (District Thatta in Sindh province), India (Belagavi and Nagpur) and Guatemala (Chimaltenango). The MNHR was initiated at each of the study sites between 2008 and 2009.

Registry administrators (RAs) are paid community health workers or nurses who identify pregnant women in their respective areas and after consent enroll them in the MNHR. Once a pregnant woman is identified, the RAs obtain basic health information at enrollment, record the date of last menstrual period, or early ultrasound report to assess gestational age, obtain a hemoglobin assessment where possible, and record the height and weight of the pregnant woman. A follow-up visit is carried out following delivery to collect information on pregnancy outcomes and health care received during delivery. Information on the study outcomes is based on medical record reviews and birth attendant and family interviews.

Stillbirth is defined using a modified WHO criterion of fetal deaths occurring at ≥20 weeks gestation (or for those without gestational age, birth weight of ≥500 g) [3].

The stillbirth rate was calculated as the number of stillbirths per 1000 births, the sum of live births and stillbirths. A macerated stillbirth was defined as a stillbirth showing signs of maceration at delivery including skin and soft-tissue changes such as skin discoloration, redness, sloughing of skin, and overriding of cranial sutures and was assumed to have occurred at least 12 h prior to delivery. The RAs were trained to recognize stillbirths with maceration using both descriptions and pictures of fetuses with this condition. Those without maceration were considered intra-partum stillbirths.

### Data analyses

For the analyses, we combined data from the Kenyan and Zambian sites as the African Region and from the Belagavi and Nagpur sites as the Indian region. Pakistan and Guatemala were considered separately.

We present stillbirth rates for the regions from 2010 to 2016; the average annual percentage decline in stillbirth rates; and distal and proximate determinants of stillbirth for these regions. The average annual percentage change in stillbirth was calculated as [(*a/b*)^(1/r)^ -1] × 100 where *a* defines the most recent (2016) stillbirth rate, *b* defines the earliest (2010) stillbirth rate and *r* defines the number of years, which for this analysis is 6 [13]. To determine the factors associated with stillbirth, we used the cumulative data for the years 2010 to 2016.

Descriptive analyses included calculation of the frequency and distribution of values. The estimates and relative risks were calculated from a Poison model with generalized estimating equations to control for cluster level effects and included terms for region, maternal age, education, parity, antenatal care, delivery location, birth weight and an interaction term for region with each of the other covariates. Interaction terms with a *p*-value > 0.2 were excluded from the multivariate model.

All data were entered and reviewed at each study site. De-identified data were transmitted with additional edits performed at the data-coordinating center, Research Triangle Institute (RTI International). All data were analyzed using SAS v.9.3 (Cary, NC).

### Ethics approval

This study was reviewed and approved by all sites’ ethics review committees Francisco Marroquin University, Guatemala; University of Zambia, Zambia; Moi University, Kenya; Aga Khan University Pakistan; KLE University’s Jawaharlal Nehru Medical College, Belagavi; Lata Medical Research Foundation, Nagpur, the institutional review boards at each U.S. partner university and the data coordinating center (RTI International). All women provided informed consent for participation in the study, including data collection and the follow-up visits.

## Results

**Fig. 1:**
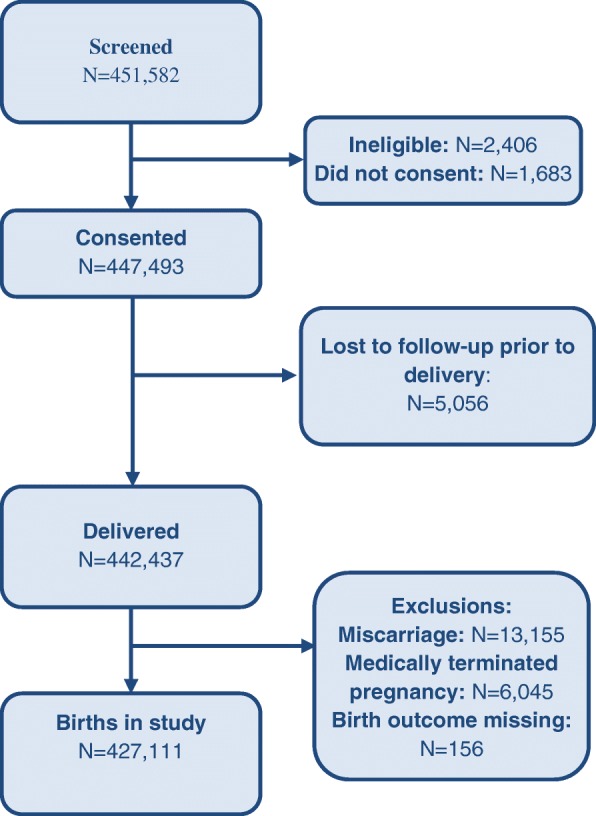
Subject Flow Diagram for Global Network Stillbirth Study, 2010–2016

During the study period from January 2010 through December 2016, 451,582 women were screened and 447,493 (99.1%) women consented to participate in the study. Delivery status was obtained for 442,437 women (98.9%) with 5056 lost to follow-up. Of these 427,111 births were included in the study. The majority of those not included had either a miscarriage or pregnancy termination (Fig. 1).

**Fig. 2:**
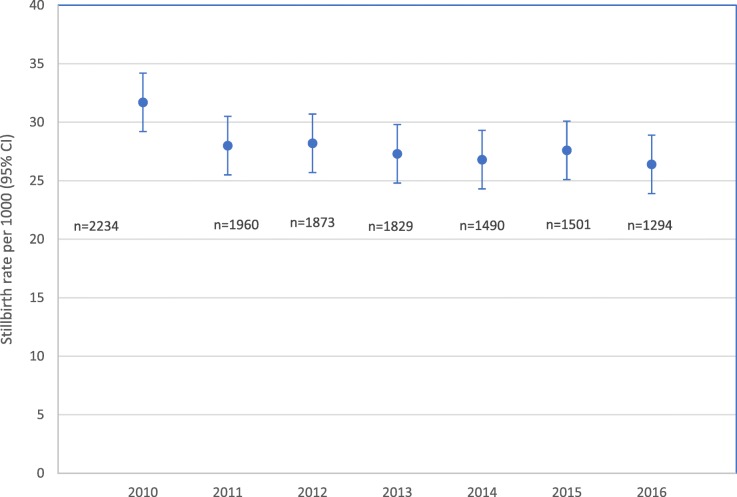
Overall stillbirth rate, Global Network sites 2010–2016. n = number of stillbirths

Of 427,111 births included, 12,181 were stillbirths, resulting in a cumulative stillbirth rate of 28.5 per 1000 births for all the regions. This rate decreased from 31.7 per 1000 births in 2010 to 26.4 per 1000 births in 2016. (Figure 2) The average annual decline in the overall stillbirth rate was 3.0%.

Table 1 provides the stillbirth rates overall and for individual regions and their 95% confidence intervals over the past seven years. Of all regions, Pakistan had the highest overall stillbirth rate of 56.9 /1000 births, and Guatemala the lowest, 19.9/1000 births. The average annual decline in the stillbirth rate for Pakistan was 3.3%. For Africa, the cumulative stillbirth rate was 21.3/1000 births which decreased from a high of 24.5/1000 in 2010 to a low of 20.5/1000 births in 2016. The average decline in the annual stillbirth rate was also 2.9%. For India the cumulative stillbirth rate was 25.3/1000 births and decreased from 31.3/1000 births in 2010 to 23.8/1000 births in 2016 giving an annual decline rate of 4.5%. The cumulative stillbirth rate for Guatemala was 19.9/1000 births and decreased from 22.7/1000 births in 2010 to 20.9/1000 birth in 2016 with an average annual decline of 1.4%. For Africa, India and Guatemala, the cumulative stillbirth rates for the various years were similar and their confidence intervals overlapped, showing an insignificant difference between the rates in these regions. However, for Pakistan, the cumulative stillbirth rates in different years were significantly higher than other regions. Overall, for all the regions, 68.6% of stillbirths were fresh (defined as no signs of maceration) indicating fetal death occurred just before or during delivery. The percentage of fresh stillbirths varied from a rate of 63.6% in Pakistan to 76.8% in Guatemala with Africa having 71.6% and India 69.0% stillbirths defined as fresh.

**Table 1:** Stillbirth rate estimates by Global Network region, 2010–2016 and rate of average annual decline

	Estimated Stillbirth Risk/1000 (95% CI)^a^				
	Total	Africa	India	Pakistan	Guatemala
Total	28.0 (26.6, 29.5)	21.3 (18.7, 24.4)	25.3 (24.0, 26.7)	56.9 (52.6, 61.6)	19.9 (17.5, 22.6)
2010	31.7 (29.5, 34.2)	24.5 (19.9, 30.2)	31.3 (28.5, 34.4)	58.4 (52.0, 65.5)	22.7 (19.5, 26.5)
2011	28.0 (25.7, 30.5)	19.0 (15.2, 23.7)	24.4 (22.0, 27.1)	62.0 (54.9, 70.1)	21.3 (17.4, 26.0)
2012	28.2 (25.6, 31.1)	24.6 (20.3, 29.9)	26.0 (24.1, 28.0)	57.7 (51.8, 64.3)	17.1 (12.5, 23.5)
2013	27.3 (25.2, 29.6)	21.5 (17.6, 26.3)	23.9 (22.1, 25.7)	62.0 (53.6, 71.8)	17.5 (14.4, 21.3)
2014	26.8 (24.8, 29.0)	20.8 (16.7, 25.8)	23.4 (20.7, 26.5)	59.2 (53.9, 65.0)	17.9 (15.1, 21.2)
2015	27.6 (25.2, 30.2)	19.2 (15.1, 24.5)	25.4 (22.5, 28.6)	52.7 (47.0, 59.0)	22.6 (18.4, 27.8)
2016	26.4 (24.4, 28.5)	20.5 (17.4, 24.1)	23.8 (20.9, 27.1)	47.7 (41.5, 54.8)	20.9 (17.4, 25.1)
Average Annual Decline (%)	3.0%	2.9%	4.5%	3.3%	1.4%

^a^Stillbirth rate estimates and 95% confidence intervals are obtained from a logistic regression model adjusting for region, year and the interaction of region by year with generalized estimating equations to control for cluster level effects

Table 2 describes the maternal characteristics in relationship to whether there was a live birth or stillbirth. Looking first at some of the population characteristics, among the live births, there are some major differences among the sites. A larger percentage of births in Africa were to women < 20 years of age compared to the other sites, while Guatemala had the largest percentage of births to women > 35 years of age. Education levels were substantially lower in Pakistan. Women with a parity of ≥3 were much more common in Pakistan, Guatemala, and Africa compared to India. Eleven percent of women in Pakistan had no prenatal care compared to 3.2% in Guatemala and < 1% in the Indian sites. Home births were common in Pakistan, Guatemala and Africa, but rare in India. The rates of preterm birth were generally similar across the sites except for Pakistan, while low birth weight rates were highest in the Asian sites.

**Table 2:** Characteristics and Delivery Outcome by Global Network Region, 2010–2016

	Africa		India		Pakistan		Guatemala	
	SB	LB	SB	LB	SB	LB	SB	LB
Births, N	2216	106,315	4415	172,594	4322	74,531	1228	61,490
Maternal age, N (%)								
< 20	533 (24.1)	24,582 (23.2)	315 (7.1)	11,682 (6.8)	175 (4.1)	3060 (4.1)	181 (14.7)	10,168 (16.5)
20–35	1468 (66.5)	75,235 (70.9)	4066 (92.2)	160,352 (93.0)	3825 (88.8)	67,432 (90.7)	789 (64.3)	44,890 (73.0)
> 35	208 (9.4)	6330 (6.0)	28 (0.6)	442 (0.3)	306 (7.1)	3863 (5.2)	258 (21.0)	6416 (10.4)
Education, N (%)								
No formal education	123 (5.6)	6062 (5.7)	675 (15.4)	22,345 (13.0)	3788 (88.0)	60,793 (81.8)	315 (25.7)	10,087 (16.4)
Primary	1018 (46.2)	48,681 (45.9)	1099 (25.0)	38,196 (22.2)	274 (6.4)	6111 (8.2)	700 (57.0)	35,557 (57.8)
Secondary+	1064 (48.3)	51,294 (48.4)	2619 (59.6)	111,363 (64.8)	244 (5.7)	7419 (10.0)	213 (17.3)	15,831 (25.8)
Parity, N (%)								
0	675 (30.5)	29,202 (27.5)	2104 (47.8)	75,531 (43.9)	872 (20.9)	13,322 (18.4)	312 (25.4)	17,350 (28.2)
1–2	721 (32.6)	40,886 (38.5)	1997 (45.4)	87,891 (51.1)	1109 (26.5)	24,387 (33.7)	332 (27.0)	23,255 (37.8)
≥ 3	815 (36.9)	36,102 (34.0)	299 (6.8)	8630 (5.0)	2199 (52.6)	34,624 (47.9)	584 (47.6)	20,879 (34.0)
≥ 1 ANC visit, N (%)	2091 (94.4)	104,967 (98.8)	4387 (99.5)	172,066 (99.9)	3581 (83.3)	65,868 (88.6)	1151 (93.8)	59,452 (96.8)
Delivery location, N (%)								
Hospital	676 (30.5)	18,210 (17.1)	3128 (70.8)	115,866 (67.2)	1437 (33.3)	23,728 (31.8)	659 (53.7)	28,647 (46.6)
Clinic	690 (31.2)	46,467 (43.7)	557 (12.6)	49,109 (28.5)	1067 (24.7)	19,491 (26.2)	26 (2.1)	1756 (2.9)
Home/Other	849 (38.3)	41,637 (39.2)	730 (16.5)	7569 (4.4)	1817 (42.1)	31,286 (42.0)	543 (44.2)	31,087 (50.6)
Preterm N (%)	975 (50.3)	11,724 (11.6)	2848 (66.6)	16,163 (9.6)	2402 (63.6)	12,105 (17.1)	481 (41.1)	5961 (9.9)
Birth weight < 2500 g, N (%)	970 (46.3)	4551 (4.3)	3089 (77.0)	27,119 (15.7)	2391 (63.0)	13,824 (18.6)	598 (52.5)	8794 (14.3)

Table 3 describes the relative risks of stillbirth and maternal characteristic, stratified by region. Overall, in each site, maternal age < 20 years of age was associated with a slightly higher stillbirth risk compared to maternal age 20–35, but only in Africa and India were the differences significant. On the other hand, age > 35 was associated with significantly higher risk of stillbirth in all sites, ranging from a RR of 1.4 in Pakistan to 2.4 in India. Compared to parity 1–2, zero parity and parity ≥ 3 were both associated with increased stillbirth risk in all sites. Compared to women with any prenatal care, women with no PNC had significantly increased risk of stillbirth in all sites, ranging from a relative risk of 1.5 in Pakistan to 4.5 in India. Except for India, where home births were associated with a 3.3 times increased risk of stillbirth compared to hospital births, both home and clinic births had lower risks of stillbirth than those in hospital.

**Table 3:** Relative Risks of Stillbirth by Global Network region, 2010–2016

	Africa	India	Pakistan	Guatemala
	SB Relative Risk^a^ (95% CI)	SB Relative Risk^a^ (95% CI)	SB Relative Risk^a^ (95% CI)	SB Relative Risk^a^ (95% CI)
Maternal age				
< 20	1.11 (1.00, 1.23)	1.13 (1.05, 1.21)	1.03 (0.91, 1.17)	1.03 (0.89, 1.20)
20–35	reference	reference	reference	reference
> 35	1.63 (1.40, 1.89)	2.39 (1.63, 3.50)	1.41 (1.27, 1.57)	2.21 (1.96, 2.49)
Education				
No formal education	0.98 (0.83, 1.16)	1.12 (0.99, 1.26)	1.73 (1.50, 2.00)	2.07 (1.74, 2.46)
Primary	1.02 (0.88, 1.17)	1.17 (1.09, 1.26)	1.31 (1.08, 1.59)	1.32 (1.11, 1.55)
Secondary+	reference	reference	reference	reference
Parity				
0	1.29 (1.14, 1.45)	1.32 (1.23, 1.41)	1.42 (1.27, 1.58)	1.26 (1.11, 1.43)
1–2	reference	reference	reference	reference
≥ 3	1.23 (1.14, 1.32)	1.38 (1.24, 1.53)	1.36 (1.26, 1.48)	1.79 (1.53, 2.08)
≥ 1 ANC visit				
Yes	reference	reference	reference	reference
No	4.92 (3.66, 6.61)	2.93 (1.46, 5.89)	1.54 (1.38, 1.71)	2.00 (1.60, 2.50)
Delivery Location				
Hospital	reference	reference	reference	reference
Clinic	0.38 (0.26, 0.55)	0.38 (0.33, 0.45)	0.88 (0.79, 0.99)	0.60 (0.40, 0.89)
Home/Other	0.52 (0.32, 0.84)	3.27 (2.54, 4.22)	0.91 (0.80, 1.03)	0.65 (0.57, 0.74)

^a^The estimates and relative risks are from a Poisson model with generalized estimating equations to control for cluster level effects and includes terms for region, maternal age, education, parity, antenatal care, delivery location and interaction terms for Region with each of the other covariates

## Discussion

In this analysis, in sites in India, Pakistan, Africa and Guatemala, from 2010 to 2016, the mean stillbirth rate declined from 31.7/1000 births to 26.4/1000 births with an average annual reduction rate of 3.0% The Every Newborn Action Plan, a global multi-partner movement to end preventable maternal and neonatal deaths and stillbirths has established a goal to reduce stillbirth rates globally to 12 or less per 1000 births by 2030 [14, 15]. If we assume a rate of 12/1000 births by 2030 and consider the Global Network SBR of 2016 as the benchmark, then the average annual rate of reduction in the stillbirth rate required would be 5.8% which is far higher than the current rate of decline.

Because our study evaluated rates of stillbirth decline in relatively small areas of five countries, we looked for other studies to compare our results. Blencowe et al. performed a large modeling exercise using thousands of data points to estimate stillbirth declines in 195 countries from 2000 to 2015 [8]. While the methodology, many of the definitions, and the years evaluated were different than our study, the overall results were relatively similar. For example, across all sites Blencowe estimated a yearly world-wide reduction in stillbirths of 2.0% while we found a yearly reduction in our sites of 3.0% Our African sites had a reduction of 2.9% while Blencowe’s African sites had an annual reduction of 1.4%. Our Asian sites had a reduction in stillbirths of 4.5% for India and 3.3% for Pakistan, while Blencowe’s Asian sites had an annual reduction of 2.2%. Our Guatemalan site had an estimated yearly reduction of 1.4% while Blencowe’ s Latin American sites had a reduction of 2.1%. Regardless of which numbers more closely approximate reality, few if any of the estimated rates of reduction are sufficient to reach the 2030 Every Newborn Action Plan goal.

The stillbirth rates in our sites are derived from population-based registries that intend to capture all pregnancy outcomes in their area. Thus, we believe these data may provide better information on stillbirth rates than data from hospital births or mathematical models. Nevertheless, the site rates reported here generally approximate the national reported rates. For example, according to a recent publication, Pakistan has the highest reported stillbirth rate in the world at 43.1/1000 births while our study reports a stillbirth rate for 2016 as 47.7/1000 births. In India, the stillbirth rates in different states reportedly range from 20 to 66/1000 births. Bellad et al. reported a stillbirth rate of 28.6/1000 births from Karnataka where one of our Indian sites is situated [16]. The stillbirth rate for Maharashtra district where Nagpur is located as reported by the State Health Department is 17.8/1000 births. The mean stillbirth rate for our Indian sites was 25.3/1000 births. The national stillbirth rate for Kenya as reported by WHO is 21.8/1000 births [17] and 25.5/1000 births for Zambia [18], which are close to our reported rates for the African region. For Guatemala, the national stillbirth rate is reported as 10/1000 births [8]. The rates from our study for Guatemala are higher than the national rate, but this difference may partially be explained by the location of our study site that is in an area where the population is primarily indigenous.

The Global Network goes to great lengths at all sites to train data collectors about using standard definitions and locating all pregnancies. Therefore, we have a high level of confidence that the rates of stillbirth presented in this paper are accurate for those regions. We are aware that in this paper we define stillbirth as having a lower gestational age limit of 20 weeks gestation while the WHO often uses a lower gestational age limit of 28 weeks gestation for international comparisons. We believe that stillbirths in the 20 to 27-week range should be counted and present those data here. The impact of this decision on the ability to compare our data with the WHO data is not clear.

Extremes of age, illiteracy, null or high parity, lack of antenatal care and place of delivery all have been documented as being independent risk factors for stillbirth and are commonly found in various LMICs [2, 5, 19]. Our study results confirm that these factors are significantly associated with stillbirth and are common in our regions. Education of women has been identified as an important factor for making timely decisions to prevent adverse maternal and fetal outcomes [19]. In Pakistan and Guatemala, low levels of education were a risk factor for stillbirth. In Pakistan, very few women had even a primary or secondary level of education. However, in Africa, nearly all women had some level of education. Women having more than two prior deliveries and nulliparas were at higher risk of having a stillbirth in all the regions as compared to women with one or two prior deliveries. The literature also suggests that women with more than two prior deliveries are at risk of developing complications during pregnancy including stillbirth [20, 21].

In nearly all the regions, receiving antenatal care during pregnancy was common. However, stillbirths were more common in women who did not receive antenatal care as compared to women who did attend at least one visit. The importance of antenatal care during pregnancy for the screening of potential medical risks such as infection, anaemia, malnutrition, and hypertension cannot be over-emphasized. Lack of quality antenatal care is a missed opportunity for the women.

Most of the stillbirths delivered at the hospitals. We assume that after developing complications in pregnancy, these women approached the hospital when it was too late to save the fetus, or the hospitals were not adequately equipped with essential services such as timely caesarian section or fetal monitoring. Studies from Zambia, Kenya, and Pakistan on the preparedness of health facilities to deal with emergencies, indicate major inadequacies in terms of staffing, training and equipment [22]. In LMICs, especially in rural areas, district hospitals are often not adequately equipped with newborn intensive care units, blood banks and laboratory support.

In addition, many stillbirths occurred in home deliveries. Information on the events which occurred prior to having a stillbirth at home was not available from our data, especially during the intrapartum period. For example, the type of health care provider, duration of labor, presentation of the fetus at the time of labor and any delivery related complications that resulted in stillbirth are often not available.

This study had a number of strengths including that it was population-based and did not rely on data only from hospital admissions. A registry administrator was responsible for identifying pregnancies as early as possible in the pregnancy and reporting the results as soon after delivery as feasible. The data were collected prospectively and five sites in six countries provided data covering more than 427,000 births. Despite the large sample size, these data were collected in relatively small, often rural areas of the individual countries and are not representative samples from the countries.

Even though many of the factors associated with stillbirth remain common to LMICs, we believe that more in-depth knowledge related to these factors will help governments and public health partners to achieve the Early Newborn Action Plan target of 12/1000 stillbirths by 2030. Understanding socio-cultural barriers for seeking care during pregnancy and childbirth may help governments and the private sector to intervene appropriately. Many adverse events occur because treatment costs are not affordable, failure to shift the woman to a facility in a timely fashion, and preference by many women for home deliveries - mostly by an unskilled attendant. With effective public private partnerships, many of these issues can be resolved.

## Conclusions

To summarize, stillbirth rates declined in all sites, but not at a sufficient rate to meet the 2030 goals of the Early Newborn Action Plan of 12/1000. Most of the distant and intermediate determinants of stillbirths were common between Africa, India, Pakistan and Guatemala. More local, in-depth information about these determinants and appropriate action to decrease their prevalence are needed to increase the pace of annual reductions in stillbirths.
